# DEVELOPMENT AND TESTING OF A MOBILE APPLICATION INTEGRATING PHYSICAL ACTIVITY AND COGNITIVE TRAINING

**DOI:** 10.1093/geroni/igz038.1430

**Published:** 2019-11-08

**Authors:** Lenora Smith

**Affiliations:** University of Alabama in Huntsville, Huntsville, Alabama, United States

## Abstract

Dementia is progressive, which causes a debility in activities of daily living, leading to bedridden and uncommunicative individuals. Evidence has shown that physical activity may protect the brain from memory impairment. Based on a previous study we conducted using consumer-based applications with people with mild cognitive impairment, we developed a mobile application called the mPACT app (mobile physical activity and cognitive training). The System Usability Scale and Usability Test Observation Coding Form were used to capture usability and verbal/nonverbal behaviors respectfully of 5 participants. Results noted an above average score for usability, with a mean score of 72.5. Observations noted that almost all participants were smiling and enjoying most of the games within the app; some had difficulty with a couple of the games. Overall, there was a positive response to the mPACT app, revisions have been made, and next steps include another beta test with people with MCI.

